# A Multidimensional Approach to Measure Poverty in Rural Bangladesh

**Published:** 2007-06

**Authors:** Abbas Bhuiya, Shehrin Shaila Mahmood, A.K.M. Masud Rana, Tania Wahed, Syed Masud Ahmed, A. Mushtaque R. Chowdhury

**Affiliations:** 1 ICDDR,B, GPO Box 128, Dhaka 1000, Bangladesh; 2 BRAC, Mohakhali, Dhaka 1212

**Keywords:** Asset index, Clothing, Education, Food, Health, Participatory rural appraisal, Poverty, Poverty measurement, Reliability, Shelter, Social exclusion, Validity, Chakaria, Matlab, Bangladesh

## Abstract

Poverty is increasingly being understood as a multidimensional phenomenon. Other than income-consumption, which has been extensively studied in the past, health, education, shelter, and social involvement are among the most important dimensions of poverty. The present study attempts to develop a simple tool to measure poverty in its multidimensionality where it views poverty as an inadequate fulfillment of basic needs, such as food, clothing, shelter, health, education, and social involvement. The scale score ranges between 72 and 24 and is constructed in such a way that the score increases with increasing level of poverty. Using various techniques, the study evaluates the poverty-measurement tool and provides evidence for its reliability and validity by administering it in various areas of rural Bangladesh. The reliability coefficients, such as test-retest coefficient (0.85) and Cronbach's alpha (0.80) of the tool, were satisfactorily high. Based on the socioeconomic status defined by the participatory rural appraisal (PRA) exercise, the level of poverty identified by the scale was 33% in Chakaria, 26% in Matlab, and 32% in other rural areas of the country. The validity of these results was tested against some traditional methods of identifying the poor, and the association of the scores with that of the traditional indicators, such as ownership of land and occupation, asset index (r=0.72), and the wealth ranking obtained from the PRA exercise, was consistent. A statistically significant inverse relationship of the poverty scores with the socioeconomic status was observed in all cases. The scale also allowed the absolute level of poverty to be measured, and in the present study, the highest percentage of absolute poor was found in terms of health (44.2% in Chakaria, 36.4% in Matlab, and 39.1% in other rural areas), followed by social exclusion (35.7% in Chakaria, 28.5% in Matlab, and 22.3% in other rural areas), clothing (6.2% in Chakaria, 8.3% in Matlab, and 20% in other rural areas), education (14.7% in Chakaria, 8% in Matlab, and 16.8% in other rural areas), food (7.8% in Chakaria, 2.9% in Matlab and 3% in other rural areas), and shelter (0.8% in Chakaria, 1.4% in Matlab, and 3.7% in other rural areas). This instrument will also prove itself invaluable in assessing the individual effects of poverty-alleviation programmes or policies on all these different dimensions.

## INTRODUCTION

Poverty, as normally defined, means that the consumption or income level of a person falls below a certain threshold necessary to meet basic needs. The most frequently-used measure of poverty is based on income or consumption proxies. Yet, “Poverty never results from the lack of one thing but from many interlocking factors that cluster in poor people's experience and definitions of poverty” (1). Poverty is the result of economic, political and social processes that interact with each other and frequently reinforce each other in ways that exacerbate the deprivation in which poor people live (2). As aptly described, “Poverty is hunger. Poverty is lack of shelter. Poverty is being sick and not being able to see a doctor. Poverty is not having access to school and not knowing how to read. Poverty is not having a job, is fear for the future, living one day at a time. Poverty is loosing a child to illness brought about by unclean water. Poverty is powerlessness, lack of representation and freedom” (3). Hence, poverty is increasingly being understood as a multidimensional phenomenon (4,5). Poverty is more than an economic condition, in which the basic necessities of life are lacking, such as food, housing, and clothing (6). Poverty is “not merely in the impoverished state in which the person actually lives but also in the lack of real opportunity, due to social constraints as well as personal circumstances, to lead valuable and valued lives” (7). Poverty is also the absence of capacity or opportunities to change the situation (6). The other elements missing from the life of the poor are good health and longevity, adequate education, access to land, credit, and other productive resources, ability to avoid and confront drastic drops in income, family and community support, justice, fair treatment, and a voice in institutions and access to opportunity (6).

Development agenda have included various strategies to reduce poverty and promote social equity. These interventions quite often are primarily focused on activities only to raise the income of the poor. Although the non-income dimensions of poverty have been recognized, they have rarely been used for assessing and monitoring the effects of various poverty-reduction programmes (8,9). Thus, the poverty-reduction programmes miss the opportunity to know the level of their success or failure in improving the non-income dimensions of poverty. One of the reasons for this could be not having an easy-to-use tool at hand. To capture the non-income dimensions of poverty, indicators other than income or consumption, e.g. indicators for education, health, access to services, and infrastructure and social support, are needed. It is against this background that the present study attempts to develop a simple tool to measure poverty in rural Bangladesh keeping its multidimensional nature in mind. This paper presents results from this exercise.

## MATERIALS AND METHODS

The process of development and validation of the poverty-measurement tool was carried out in two phases. The first phase involved development of the poverty-measurement tool that included testing the reliability and validity of the instrument. A wealth ranking of households using participatory rural appraisal (PRA) methods (4) was carried out by informed villagers at the end of the first phase. The socioeconomic groups identified by the villagers through this exercise were then used for determining the corresponding cut-off points of the total poverty score. In the second phase, the poverty-measurement tool was applied to a larger population in rural Bangladesh to estimate the level of poverty in those areas.

### Development of tool

For our purpose, poverty has been viewed as inadequate fulfillment of basic needs, such as food, clothing, shelter, health, education, and social involvement. The initial step was to enlist relevant items that represent the various dimensions of basic needs. A panel of informed professionals then judged the list of items to assess their relevance. When the panel reached a consensus on the relevance of the items, a data-collection instrument was developed with the listed items, which was subsequently field-tested. After the field-testing, four items per dimension were selected for inclusion in the final instrument. All the items had three answers which were assigned three different scores representing the degree of poverty—3 for the highest level of poverty and 1 for the lowest. Thus, the total poverty score could have a maximum value of 72 and a minimum value of 24. The higher the score the poorer would be the household. The data-collection instrument is presented in the Appendix.

### Data collection

In the first phase, 10 interviewers, with a minimum of 14 years of education, were trained to collect information from all 129 households of a selected village in Chakaria upazila under Cox's Bazar district in February 2002. This was repeated in the same population by the same interviewers in the same households after four weeks of initial data collection to test the reliability of the data-collection instrument. A training manual was prepared that explained the objectives of the survey and included definitions of some key terms used in the questionnaire. The interviewers used this manual as a reference guide.

Seventeen participants who had a comprehensive knowledge of the whole village were selected to conduct the PRA session. The participants were asked to divide the households in the whole village into groups based on the poverty status of the households. The participants were asked to stratify the households according to five categories: well-off, moderately well-off, not so well-off, poor, and very poor.

The second phase involved data collection from 10,612 households of 10 villages in Matlab upazila under Chandpur district as part of the joint BRAC-ICDDR,B Project activities during April-September 2002 and from 2,405 households in 12 rural sites as a part of data collection by the Bangladesh Health Equity Watch (10) during May-July 2003. Interviewers were trained in the same manner as before to carry out the second phase of data collection. Data were collected from the household head or another informed household member in the absence of the household head.

### Analysis

The first step of data analysis was devoted to the development of the tool, which also included testing the reliability and validity of the instrument. The test retest, split-half reliability, and Cronbach's Alpha test were carried out using the SPSS software to test validity and reliability (11–13). A poverty score for each household was calculated by adding the scores from each of the six dimensions. In the second step, the poverty scores of the households were compared with the wealth ranking obtained by the PRA and other traditional indicators, such as amount of land and various other assets owned by the household, and occupation of the household head. In addition, the relationship of poverty index with asset index was examined by bivariate analysis. Poverty index and asset index were calculated using principal component analysis (14–17). Correlation and analysis of variance techniques were used in examining the relationship between the traditional indicators and the poverty scores.

## RESULTS

### Reliability

Table 1 presents the reliability estimates for all the items that were included in the instrument and also for the overall scale. The reliability estimate for the test-retest method for the overall scale was 0.85, which is considered to be a satisfactorily high coefficient of stability. It means that the scale is valid and yields consistent results over time and is capable of producing results that can be considered stable in 85% of the time.

**Table 1:** Reliability coefficients for the subscales and the whole scales in the two rounds (n=129)

Dimension	Round	Reliability coefficient—split-half	Index of reliability—Cronbach's alpha	Reliability coefficient— test-retest
Education	1	0.41	0.67	0.742
	2	0.41	0.68	
Health	1	0.23	0.38	0.500
	2	0.08	0.10	
Food	1	0.29	0.51	0.722
	2	0.38	0.47	
Housing	1	0.06	0.28	0.828
	2	0.08	0.29	
Clothing	1	0.45	0.61	0.685
	2	0.51	0.64	
Social participation	1	0.33	0.57	0.713
	2	0.24	0.62	
Scale as a whole	1	0.59	0.80	0.854
	2	0.52	0.75	
Standard deviation for the whole scale	1	7.15	7.15	
	2	6.57	6.57	-

The split-half reliability coefficient, which assesses the consistency of items within a measurement tool, of 59% (52% for the second round) offered reasonable support for internal consistency. A more common measure of internal consistency—Cronbach's alpha (where the coefficient represents the average of all possible split-half estimates)—was found to be satisfactory with an estimate of 0.80 (0.75 for the retest survey). The value for alpha should ideally exceed 0.70, while value in excess of 0.90 might suggest that some items are redundant (18).

The test-retest, split-half, and Cronbach's alpha were also calculated for each of the six dimensions separately. The reliability coefficient for the split-half test did not come out to be very satisfactory for all the items. The highest coefficient was 0.45 for clothing, and the lowest was 0.06 for housing. The reason for this low reliability estimate was that some items were in some cases negatively related with each other, and in some cases, the correlation between the items was low. This indicates that there is a scope to modify the scale further to reduce this inconsistency.

The test-retest method, however, showed quite high reliability for all the components. The highest coefficient was 0.83 for housing, followed by education with a coefficient of 0.74.

### Poverty score and other measures

As mentioned earlier, the validity of the scale was also examined by comparing the poverty scores with the PRA wealth-ranking results. The criteria that the villagers used for wealth ranking were mainly ownership of land, status of job, presence of day labourer or beggar in the household, and having members of the household living abroad.

Table 2 presents the mean total and subscale poverty score by the ‘wealth ranking’ of the households. According to the wealth ranking, 5% of the households were in the well-off category (most better-off) and 33% in the very poor category (poorest). The mean poverty score was lowest for the well-off category and highest for the very poor category. The relationship between the wealth-ranking categories and the mean poverty score was linear and negative. The pattern of the relationship in the two rounds was similar. The relationship between the mean scores and the wealth-ranking categorization was also linearly negative for all the subscales. The households in the lowest category by wealth always had the highest poverty score for all the subscales.

**Table 2:** Mean household poverty score in the two rounds by household socioeconomic status measured by participatory rural appraisal wealth ranking, Chakaria, 2002

Dimension	Round	Well-off	Moderately well-off	Not so well-off	Poor	Very poor	p value	Total score
Education	1	7.0±2.1	7.1±1.7	7.7±2.1	9.1±2.0	9.4±1.9	0.00	8.7±2.1
	2	6.7±2.3	6.7±1.8	7.6±1.6	8.5±2.1	9.2±2.1	0.00	8.3±2.2
Health	1	5.5±1.8	5.9±1.3	6.0±1.1	6.5±1.2	7.0±1.4	0.00	6.5±1.3
	2	5.5±1.1	5.9±0.9	5.8±0.9	6.1±0.9	6.6±1.1	0.00	6.2±1.0
Social								
participation	1	5.8±1.2	7.5±1.9	7.6±2.2	8.5±1.9	9.4±1.7	0.00	8.4±2.1
	2	5.8±0.8	8.0±1.7	8.0±2.3	8.6±1.9	9.7±2.0	0.00	8.7±2.1
Shelter	1	6.0±1.1	7.1±1.3	7.5±1.2	8.0±1.2	8.5±1.2	0.00	7.9±1.3
	2	6.5±0.8	6.5±1.1	7.6±1.5	8.2±1.2	8.6±1.4	0.00	8.0±1.4
Food	1	6.2±1.0	5.9±1.0	6.7±1.3	7.3±1.2	8.1±1.4	0.00	7.3±1.4
	2	5.8±1.7	6.0±1.1	6.7±1.0	7.2±1.0	8.1±1.3	0.00	7.2±1.3
Clothing	1	4.3±0.8	4.3±0.6	4.7±0.9	5.5±1.3	6.6±1.7	0.00	5.6±1.5
	2	4.0±0.0	4.2±0.4	4.8±1.1	5.4±1.2	6.6±1.4	0.00	5.5±1.4
Total score	1	34.8±2.9	37.9±4.9	40.1±5.7	45.0±5.0	49.0±6.3	0.00	44.3±7.0
	2	34.3±4.2	37.3±3.2	40.6±5.7	44.1±4.6	48.7±5.4	0.00	43.9±6.5
N		6	13	22	46	42		129

### Ownership of land and occupation

In an attempt to examine the correlation between the traditional indicators of socioeconomic status and the obtained poverty score, ownership of land, and occupation were combined to classify a household into three socioeconomic categories—high, medium, and low. Households depending on manual labour were considered to be in the lowest socioeconomic group, irrespective of ownership of land; households owning less than 50 decimals of land and not depending on manual labour were in the middle group; and the households owning more than 50 decimals of land with no manual labour were considered to be in the highest socioeconomic group.

Table 3 shows that the total poverty score was negatively related to the household socioeconomic status as defined by the land-occupation criterion. According to the poverty scale, the lowest socioeconomic group scored 47.5, on average, in the poverty scale, the middle group an average of 43.2, and the highest socioeconomic group (well-off) 36.8. The tool clearly provides a poverty scale consistent with the socioeconomic status ranking where the score of the scale gradually decreases with an increase in the socioeconomic status defined by ownership of land and occupation. The results were almost similar in the first and the second round, implying that the poverty scores were reliably consistent.

Table 3 also presents the poverty scores by land-occupation classification for all subscales, such as shelter, food, clothing, education, health, and social participation. The subscale score totals were negatively related to the socioeconomic status of the household measured by the land-occupation criterion.

**Table 3:** Mean household poverty score in the two rounds by household socioeconomic status measured by ownership of land and occupation, and asset score, Chakaria, 2002

Dimension	Round	Upper	Middle	Low	p value
Education	1	6.8±1.7	8.7±1.9	9.3±2.0	0.00
	2	7.1±2.4	7.8±2.0	8.9±2.0	0.00
Health	1	5.9±1.4	6.4±1.4	6.7±1.2	0.03
	2	6.0±1.1	5.9±1.0	6.5±1.0	0.01
Social participation	1	7.1±2.1	7.9±1.9	9.2±1.8	0.00
	2	8.3±2.1	7.4±1.9	9.6±1.9	0.00
Shelter	1	6.5±1.1	7.7±1.2	8.5±1.1	0.00
	2	6.7±1.5	8.6±1.5	9.3±1.2	0.00
Food	1	6.2±1.1	7.1±1.5	7.7±1.3	0.00
	2	6.1±1.3	6.9±1.1	7.7±1.3	0.00
Clothing	1	4.4±0.7	5.3±1.6	6.1±1.4	0.00
	2	4.5±0.9	4.9±1.3	6.2±1.4	0.00
Total score	1	36.8±4.6	43.2±6.5	47.5±5.6	0.00
	2	38.6±6.9	41.5±4.9	48.1±5.4	0.00
N	1	22	41	66	
	2	16	45	68	

### Ownership of assets

**Fig. 1:**
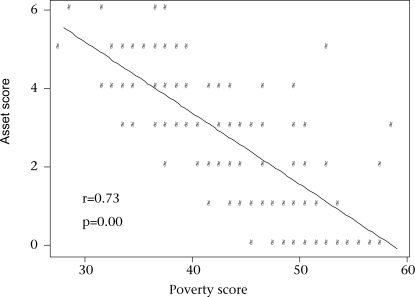
Scatter plot of total poverty score and asset-based score obtained by simple summation, Chakaria, 2002

**Fig. 2:**
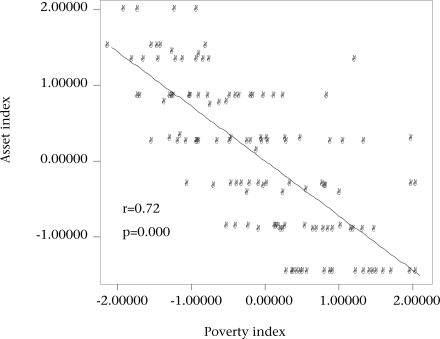
Scatter plot of poverty index and asset index obtained through principal component analysis, Chakaria, 2002

Ownership of assets was used as an indicator of the socioeconomic status of the household. The questionnaire included information on asset ownership of the households. In total, six assets, such as TV, radio, khat (bed), quilt, table/chair, and clock, were included. For our analysis, a household scored a maximum of six if it had all 6 assets and 0 if it had no assets. Fig. Figure 1 presents the scatter diagram depicting the linear and negative or inverse relationship between the asset-based score and the poverty score. A comparison of the results of the poverty scale was also made with that of the widely-used asset index. Using weights derived from the principal component analysis, an asset index was calculated for each of the households based on the information on ownership of the above-mentioned six assets. A similar procedure was followed to derive a poverty index from the poverty scale. Fig. Figure 2 presents the scatter diagram, which again depicts a linear and negative relationship between these two indices.

### Level of poverty in Matlab and in other rural areas as measured by poverty score

In the second phase of the analysis, the poverty-measurement tool was applied in Matlab and in other rural areas of Bangladesh. The mean and median poverty scores for Matlab were 41.15 and 41 respectively with a standard deviation of 5.67. For the other rural sites of 12 districts in Bangladesh, the mean and median poverty scores were 41.41 and 41 respectively with a standard deviation of 7.22. Table 4 presents the distribution of households by socioeconomic status in Matlab and in other rural areas, using cut-off points, which match the wealth ranking in Chakaria.

**Table 4:** Distribution of households in Matlab (for 2002) and in other rural sites in 12 districts (for 2003) by poverty score classified by cut-off points corresponding to wealth ranking in Chakaria

Poverty score	% of households in Matlab (n=10,612)	% of households in rural sites of 12 districts (n=2,405)
≤35 (well-off)	15.3	22.1
35-38 (moderately well-off)	17.6	15.6
38-41 (not so well-off)	21.3	16.4
41-44 (poor)	19.6	14.4
44+ (very poor)	26.2	31.5
Total	100.0	100.0

Table 4 shows that one-fourth (26.2%) of the households in Matlab and one-third (31.5%) of the households in other rural areas were classified as very poor in terms of the overall poverty score. On the other hand, 15.3% of the households in Matlab and 22.1% of the households in other rural areas were well-off. Almost 59% of the households in Matlab and 46.3% of the households in other rural areas belonged to the middle-socioeconomic groups.

### Absolute level of poverty

Table 5 presents the percentage of households considered to be in absolute poverty in terms of the non-fulfillment of basic needs in Chakaria, Matlab, and other sites studied. To portray the worst-case scenario or level of absolute poverty, one item of the four in each of the dimensions was chosen as the measure of extreme poverty. The choices are elaborated below.

**Table 5:** Percentage of households in absolute poverty in various dimensions, Chakaria (2002), Matlab (2002), and other rural sites (2003)

Dimension	Chakaria (n=129)		Matlab (n=10,612)		Rural sites of 12 districts (n=2,405)	
	No.	%	No.	%	No.	%
Food						
How frequently it so happened during the last 12 months that at least some household members could not have three (breakfast, lunch, dinner) meals (rice/*ruti*) a day due to shortage of food?						
Quite commonly (four or more days in a month)	10	7.8	306	2.9	72	3.0
Shelter						
Does this household own any shelter anywhere?						
No land, no house	1	0.8	153	1.4	89	3.7
Clothing						
Do all the members of the household have three or more sets of clothes?						
Less than half have	8	6.2	882	8.3	482	20
Health						
How frequently the household members on average suffer from illness or ill-health?						
Quite frequently (once or more a month)	57	44.2	3,859	36.4	941	39.1
Education						
How common is writing ability of household members aged 10 years and above?						
None can write	19	14.7	849	8.0	403	16.8
Social participation						
How intensely any member of the household participates in *samajik*/community activities?						
Not active at all	46	35.7	3,028	28.5	536	22.3

Among the four items included to assess the educational dimension, the current level of writing ability of the household members aged over 10 years was considered most appropriate especially with the recent upward trend of school enrollment in rural Bangladesh. It was seen from Table 5 that 14.7% of the households in Chakaria, 8.0% in Matlab, and 16.8% in other sites had not a single member above the age of 10 years who could write. These households were considered to be absolute poor in terms of education.

In terms of health, the frequency of illness of the household members was considered an appropriate representation of the health status of the household members. In Matlab, 36.4% of the households reported that they had at least one episode of illness per month, while this proportion was 44.2% in Chakaria and 39.1% in other rural sites (Table 5).

For social participation, households with members without any involvement in social activities were chosen to be the appropriate indicator as it reflects the extent of isolation of the members of a household from the mainstream rural society. It was observed that 28.5%, 35.7%, and 22.3% of the households in Matlab, Chakaria, and other sites respectively have not been participating in any social activities (Table 5).

In terms of shelter, household with no shelter of its own was the chosen indicator. The members of such households lived in the land or houses of others. It was seen from Table 5 that 0.8% of the households in Chakaria, 1.4% in Matlab, and 3.7% in other sites did not own a shelter of their own.

The observation on the frequency of starvation was chosen as the indicator that would reflect the level of absolute poverty in terms of food intake. It was reported that 7.8% of the households in Chakaria, 2.9% in Matlab, and 3.0% in other sites had members who were half-fed at least once a week (Table 5).

For clothing, the observation on the number of clothes owned by the members of the household was the chosen indicator. 6.2% of the households in Chakaria, 8.3% in Matlab, and 20% in other rural sites were classified as absolute poor as most members of these households did not even own three sets of clothing (Table 5).

When all the different dimensions of absolute poverty were considered collectively, a striking difference compared to the other sites was observed in Chakaria. Although this difference can be attributed to the small sample size, it is a notable finding. It was observed that 32% of the households in Matlab and 37% in other rural sites were considered non-poor compared to a low 9.3% non-poor households in Chakaria. On the other extreme, the percentage of households that were poor in all the dimensions was much lower in Matlab and other rural sites compared to Chakaria (Table 6).

**Table 6:** Percentage of households in various levels of absolute poverty, Chakaria (2002), Matlab (2002), and other rural sites (2003)

Absolute poverty	Chakaria	Matlab	Rural sites of 12 districts
	(n=129)	(n=10,612)	(n=2,405)
Non-poor	9.3	31.5	37.0
Poor in 1 dimension	27.9	39.5	35.6
Poor in 2 dimensions	18.6	17.6	17.8
Poor in 3 dimensions	20.9	7.6	5.8
Poor in 4 dimensions	11.6	2.9	3.0
Poor in 5 dimensions	9.3	0.7	0.8
Poor in 6 dimensions	2.3	0.1	0.0
Total	100.0	100.0	100.0

## DISCUSSION

The present study evaluates the poverty-measurement tool that incorporates the non-income dimensions of poverty and provides evidence for its reliability and validity. The test-retest method suggests that the scale is capable of producing results that are consistent or stable over time. The values of Cronbach's alpha were acceptable and suggest that the items that comprise the overall instrument perform as consistent measures and are probably measuring the same construct. The poverty-measurement tool did not perform that well in terms of the split-half reliability estimate or in the item responses. This probably reflects the scope for further modifications of the scale for better accuracy. In the second step of data analysis, the poverty-measurement tool was compared with wealth ranking (using the PRA method) and other traditional indicators. The association of the constructed poverty scale with the traditional indicators of socioeconomic status and the wealth ranking obtained from the PRA were consistent. A statistically significant inverse relationship was observed in all the cases. This provides justifiable evidence of the reliability and validity of the poverty-measurement scale. The scale also contains the additional feature of measuring absolute poverty. Taking the most extreme categories in each of the six dimensions of the scale as indicators of absolute poverty, one can easily find out the ultra poor group in a certain community. While analyzing the level of absolute poverty (Table 5) in the current study, health was found to be the dominant dimension of poverty, followed by social exclusion, clothing, education, food, and shelter. In addition, due to the simple nature of the instrument, the data-collection process is straightforward, and implementation of the module takes 30-40 minutes.

A concern relating to the relative concept of poverty is that the generalizability of the scale might have been compromised as the items in the subscales were selected on the basis of a country-specific package of basic needs and on the consensus of experts. One of the advantages of the poverty measure being based on the broader concept of basic needs is that it is possible to know what aspects of life plays an important role in the concept of poverty. This instrument will also prove itself invaluable in determining the individual effects of poverty-alleviation programmes or policies on all these different dimensions.

## Appendix: Poverty-measurement tool

### Lack of education

What is the level of education the head of the household had passed?

**Table:**

Never been to school	3
Primary or less	2
Above primary	1

What is the level of education the father of the household head had passed?

**Table:**

Never been to school	3
Primary or less	2
Above primary	1

How common is the reading ability of the household members aged 10 years and above?

**Table:**

Half or more can read	1
Less than half can read	2
None can read	3

How common is the writing ability of the household members aged 10 years and above?

**Table:**

Half or more can write	1
Less than half can write	2
None can write	3

### Lack of health

How frequently do the household members on average suffer from illness or ill-health?

**Table:**

Quite frequently (once a month or more)	1
Not so frequently (once in 3 months or less)	2
Not at all/very rarely (once a year or less)	3

In the case of illness of the household members, how often an allopathic doctor is contacted?

**Table:**

Most of the time (in more than half of the cases)	1
Now and then (half or less of the time)	2
Once in a while or never	3

In the case of diarrhoeal illness of the household members, how frequently ORS [Oral rehydration solution] is administered at home?

**Table:**

Most of the time	1
Sometimes (half or less of the time)	2
Once in a while or never	3

How common is it to wash hands with soap after defaecation among the household members?

**Table:**

Most of the time (more than half the time)	1
Now and then (half or less of the time)	2
Once in a while	3

### Social participation

How intensely any member of the household participates in the *samajik*/community activities?

**Table:**

Highly actively	1
Not so actively	2
Not active at all	3

To what extent the members of this household can expect to receive financial help and social support from relatives/friends?

**Table:**

Full support	1
Some support	2
No support at all	3

How often do you/any member of the household visit Dhaka/Chittagong city?

**Table:**

Quite frequently (once or more a month)	3
Now and then (three or more times a year)	2
Very rarely (less than three times a year)	1

How often any member of the household reads newspaper or listens to radio or watches television?

**Table:**

Quite frequently (almost everyday)	1
Not so frequently (once a month or more than once in three months)	2
Rarely (once in three months or less)	3

### Lack of shelter

Does this household own any shelter anywhere?

**Table:**

Has house (land and house)	1
Has land no house or has house no land	2
No land no house	3

What is the roof of the largest dwelling made of?

**Table:**

Pucca	1
Tin/tiles	2
Straw/polythene	3

How many *ghars* (structure/room) the house has?

**Table:**

One	3
Two	2
Three and more	1

What type of latrine does the household own?

**Table:**

Sanitary	1
Non-sanitary	2
Does not own any	3

### Lack of food

How frequently it so happened during the last 12 months that at least some household members could not have three (breakfast, lunch, dinner) meals (rice/*ruti*) a day due to shortage of food?

**Table:**

Quite commonly (four or more days in a month)	3
Now and then (once or less in a month)	2
Very rarely	1

How commonly meat is cooked in this household?

**Table:**

Quite commonly (15 days or more in a month)	1
Now and then (less than 15 days a month)	2
Not at all	3

How commonly lentil or any kind of legume is cooked in this household?

**Table:**

Quite commonly (almost everyday)	1
Now and then (four or less times a month)	2
Not at all/rarely	3

How frequently milk is consumed?

**Table:**

Quite commonly (almost everyday)	1
Now and then (four or less no. of days in a month)	2
Not at all/very rarely	3

### Lack of clothing

How frequently it happened during the last 12 months that at least some household members had to wear torn/second-hand clothes due to shortage of clothing?

**Table:**

Most days	3
Now and then	2
Not at all	1

Do all the members of the household have three or more sets of clothes?

**Table:**

All have	1
The majority have	2
The majority do not have	3

How frequently during the last year at least some household members had to live with clothes received as donation, such as *zakat* or the like?

**Table:**

Most of the time	3
Now and then	2
Not at all	1

What proportion of the household members (walking children and above) has sandals/ shoes?

**Table:**

Most of them	1
Some of them	2
None of them	3
